# Independent Associations of Stroke and Sarcopenia-Related Phenotype with Lower-Limb Strength and Gait: A 1:2 Matched Cohort Analysis

**DOI:** 10.3390/jcm15207779

**Published:** 2026-10-09

**Authors:** Sunghoon Shin, Yungon Lee

**Affiliations:** 1 Neuromuscular Control Laboratory, School of Kinesiology, Yeungnam University, Gyeongsan 38541, Gyeongbuk, Republic of Korea; 2 Research Institute of Human Ecology, Yeungnam University, Gyeongsan 38541, Gyeongbuk, Republic of Korea

**Keywords:** stroke, sarcopenia-related phenotype, gait, lower-limb muscle strength, gait variability, older adults, matched cohort study, Mahalanobis distance matching

## Abstract

**Background/Objectives:** Whether stroke and sarcopenia relate to the same components of lower-limb function is unclear. We estimated each exposure’s mutually adjusted association with strength, gait performance, and gait variability, and their interaction. **Methods:** Thirty-three stroke survivors were matched 1:2 to 66 controls (n = 99) and classified into operational sarcopenia-related phenotype groups by AWGS 2019 muscle-mass and gait-speed cut-offs, not diagnoses. Strength, spatiotemporal gait, and gait variability were analysed in rank-based additive MANCOVAs (Pillai’s trace; permutation within matched sets; Holm correction across domains). Further runs added gait speed, removed the phenotype, added weight, or substituted ASM/Ht^2^. **Results:** Stroke was associated with strength (Pillai’s V = 0.240, Holm *p* = 0.003) and gait variability (V = 0.096, Holm *p* = 0.044; not significant after adjustment for weight), but not with gait performance (Holm *p* = 0.133) unless the phenotype was removed (V = 0.194, Holm *p* < 0.001). The phenotype was associated with all three domains (Holm *p* ≤ 0.008) through its slow-gait group; the low-ASM group and ASM/Ht^2^ showed no associations. At a given gait speed, neither exposure was associated with gait (Holm *p* ≥ 0.247). Several judgements varied across matched datasets. **Conclusions:** In this cohort, lower-limb function followed strength and walking performance more closely than muscle mass. The stroke association with strength held across matched datasets and limb rules, and neither exposure’s gait association was detectable at a given walking speed. No interaction was detected, and the estimates are imprecise, particularly for strength. These are conditional cross-sectional associations.

## 1. Introduction

Stroke and sarcopenia are among the most common and disabling conditions affecting older adults, and their downstream effects on neuromuscular function and mobility overlap considerably [1,2,3]. Stroke causes focal neurological injury that disrupts descending motor control and commonly results in hemiparesis, disuse atrophy, and a decline in muscle quality [4,5]. These changes are not confined to the paretic limb: relative to age-matched controls, deficits in muscle size and strength are present in the non-paretic limb as well [5]. Sarcopenia, defined by loss of muscle mass together with reduced muscle strength or physical performance [6,7], independently impairs ambulation, increases fall risk, and is associated with poorer functional recovery after stroke [3,8].

Despite a well-established literature on each condition separately, how the two relate to neuromuscular and gait outcomes when both are present has been less thoroughly characterised. Three considerations motivated this study. First, sarcopenia is common in stroke rehabilitation populations, with reported prevalence approaching 50% and attributed to immobility, inflammation, and wasting [3,9,10]; a recent scoping review of 26 studies noted increasing methodological heterogeneity across this literature [11,12]. Second, the two conditions have been proposed to interact, with stroke amplifying pre-existing low muscle mass and sarcopenia delaying neuroplastic recovery [3,13]. Third, muscle strength, spatiotemporal gait performance, and gait variability depend on partially distinct neural and musculoskeletal substrates, so the two conditions need not act on the same aspects of lower-limb function.

We therefore examined stroke and a sarcopenia-related phenotype as two exposures acting on three domains of lower-limb function. Because grip strength was not available and gait speed was obtained from a 6-min walk rather than a 6-metre usual-pace test, participants were assigned to operational sarcopenia-related phenotype groups rather than to AWGS 2019 diagnostic categories [14]; the distinction is set out in Section 2.2 and is maintained throughout.

The primary aim was to estimate the association of each exposure, adjusted for the other, with (i) isometric strength of seven lower-limb muscle groups, (ii) spatiotemporal gait performance, and (iii) gait variability. Because gait speed enters the phenotype definition, we also examined these associations at a given gait speed, without adjustment for the phenotype, and with a muscle-mass index in place of the phenotype. A secondary, exploratory aim was to examine whether the two exposures interact within each domain.

## 2. Materials and Methods

### 2.1. Participants and Matching

Stroke participants were recruited under the same institutional ethics approval and laboratory programme as earlier work from our group on post-stroke gait [15,16]. Thirty-three stroke survivors were identified and all were analysed.

Control candidates were drawn from institutional cohorts of community-dwelling older adults. Of 629 screened records, 367 remained after exclusion for a history of stroke or hemiparesis, age below 50 years, or incomplete strength or gait data. The pool was then reduced to the level of the person rather than the record. Several participants had been assessed on more than one occasion within the same source cohort; for these individuals a single record was retained, defined as the observation with the greatest age at assessment. Because the pool was assembled from more than one cohort, the same individual could also appear in more than one of them; such individuals were identified by exact agreement on name, sex, and height together with a date of birth, and a single record was retained for each. One further pair met the anthropometric criteria but not the date-of-birth criterion; because the two records could not be confirmed as the same person, one of them was excluded from selection rather than merged. These steps left 259 eligible candidates.

**Figure 1:**
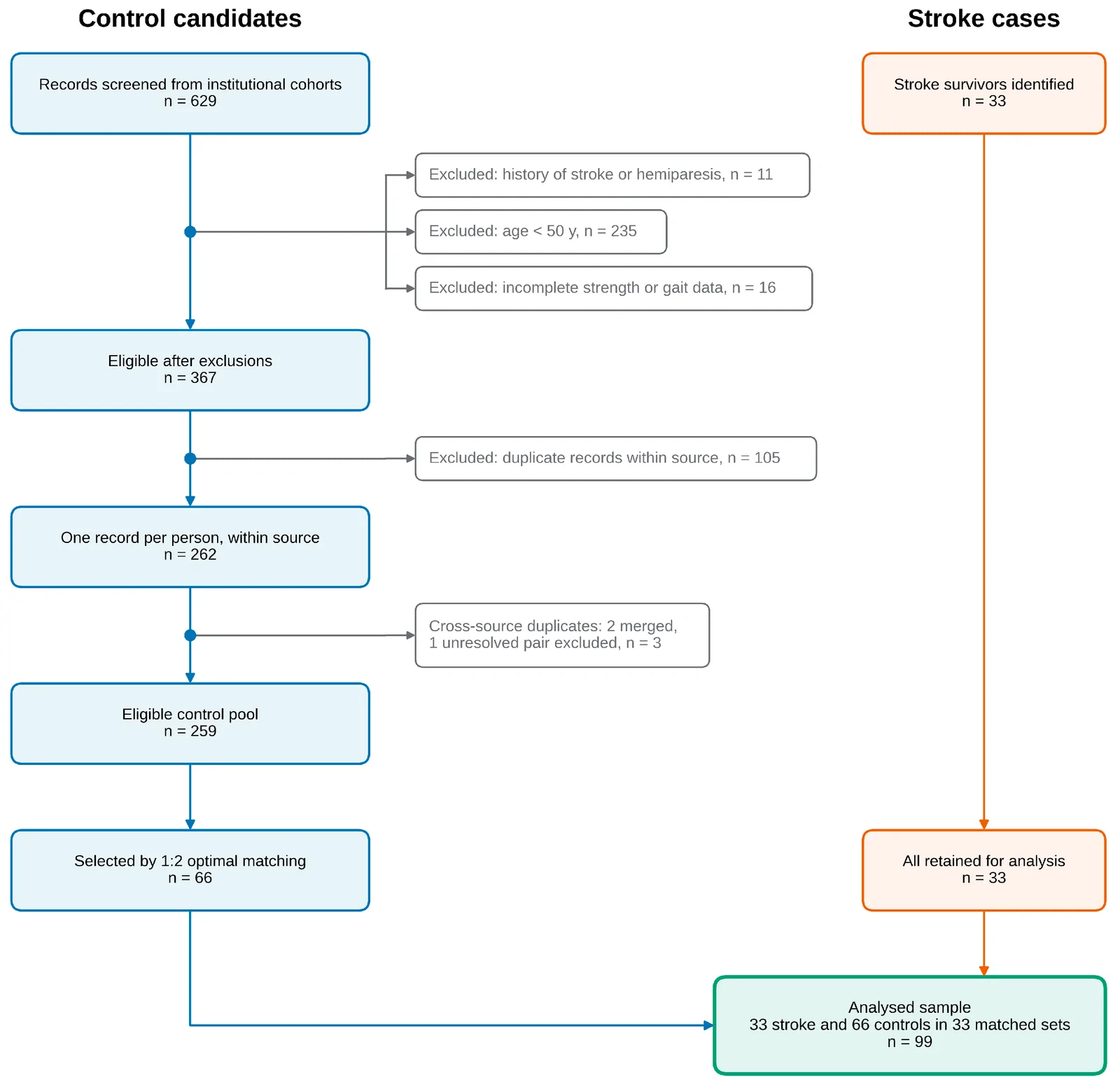
Assembly of the study cohort. Thirty-three stroke survivors were identified and all were analysed. Of 629 screened control records, 367 remained after exclusion for stroke history, age below 50 years, or incomplete data; reduction to one record per person left 262, and two cross-source merges together with one unresolved pair excluded from selection left 259 eligible candidates, from which 66 controls were selected by 1:2 optimal matching. The analysed sample is 33 stroke survivors and 66 controls in 33 matched sets (n = 99).

Each stroke case was matched to two controls by global optimal assignment (Supplementary Methods S1). Sex was matched exactly, and an age caliper of ±7 years was imposed. Within these constraints, the assignment cost combined the Mahalanobis distance on age, height, and weight, computed with the covariance matrix of the eligible control pool, with a signed age term that favoured older controls. The signed age term was included because the available pool was younger than the stroke cases, so that distance minimisation alone would have produced a control group younger than the cases; it was weighted so that the total age of the selected controls was maximised first and the total Mahalanobis distance minimised second. Matching was performed without replacement at the level of the individual, and the assignment was solved globally (linear sum assignment) rather than sequentially, so that no case could pre-empt controls needed by another; the procedure is deterministic and uses no random numbers. All 66 matched controls fell within the caliper, and each appears exactly once. The controls came from five institutional cohorts (Supplementary Table S3). Covariate balance after matching is reported in Table 1, and the assembly of the cohort is shown in Figure 1.

The dependence of observations within matched sets was taken into account in the permutation inference (Section 2.5).

Clinical characterisation was available for 30 of the 33 stroke participants (Supplementary Table S1); for the remaining three the clinical intake record was not available, and their clinical variables are reported as missing throughout. The cohort was predominantly in the chronic stage post-stroke (mean 106.7 ± 78.0 months), comprising 24 ischaemic and 6 haemorrhagic strokes with 20 left- and 10 right-sided hemipareses. Eight of 30 participants used a walking aid. Global mobility (Rivermead Mobility Index 12.0 ± 1.9 of 15) and cognitive function (Mini-Mental State Examination 24.2 ± 4.1 of 30) were in the moderately preserved range. A formal index-stroke severity measure was not available.

### 2.2. Sarcopenia-Related Phenotype Classification

Participants were assigned to phenotype groups using the muscle-mass and physical-performance components of the 2019 consensus criteria of the Asian Working Group for Sarcopenia [14]: appendicular skeletal muscle mass index (ASM/Ht^2^, multifrequency bioelectrical impedance analysis) and gait speed. Because the operationalisation used here departs from the AWGS 2019 [14] diagnostic algorithm (grip strength was not available, and gait speed was derived from a 6-min walk rather than a 6-metre usual-pace test), participants are described throughout as belonging to sarcopenia-related phenotype groups rather than as having or not having sarcopenia. The term is abbreviated to “phenotype” hereafter.

ASM was determined by multifrequency bioelectrical impedance analysis (InBody 370S; InBody Co., Ltd., Seoul, Republic of Korea), which has been validated against dual-energy X-ray absorptiometry in Asian older adults [17,18]. Participants were assessed barefoot and in light clothing in the standardised standing posture defined by the manufacturer’s protocol, after a minimum 4-h fast and bladder voiding. ASM was normalised by squared height (ASM/Ht^2^, kg/m^2^), and the AWGS 2019 sex-specific cut-offs were applied: below 7.0 and below 5.7 kg/m^2^ for men and women, respectively [14]. Gait speed for classification was the mean 6-min walk speed (Section 2.4), with the physical-performance cut-off set at 1.0 m/s.

Three phenotype groups were defined:

1. Robust: ASM/Ht^2^ at or above the sex-specific cut-off and gait speed at or above 1.0 m/s.
2. Slow gait with preserved muscle mass: ASM/Ht^2^ at or above the sex-specific cut-off but gait speed below 1.0 m/s.
3. Low ASM: ASM/Ht^2^ below the sex-specific cut-off, regardless of gait speed.

Gait speed enters the phenotype definition. It was therefore excluded from the gait performance block in the primary analysis, so that no variable both defines the exposure and serves as an outcome for it. Its role was examined in two further runs, with gait speed as a covariate and, with the phenotype replaced by ASM/Ht^2^, as an outcome (Section 2.5).

### 2.3. Muscle Strength Assessment

Isometric maximal voluntary contraction strength was evaluated for seven lower-limb muscle groups bilaterally: hip flexion (iliopsoas), hip extension (gluteus maximus), hip abduction (gluteus medius), knee flexion (hamstrings), knee extension (quadriceps), ankle plantarflexion, and ankle dorsiflexion. A portable handheld dynamometer (microFET2, Hoggan Scientific, Salt Lake City, UT, USA), validated for isometric strength evaluation in elderly and clinical populations [19,20], was used by a single trained examiner. Hip flexion and knee extension were tested seated with the hip and knee at 90°; hip extension, hip abduction and knee flexion were tested prone; ankle plantarflexion and dorsiflexion were tested supine with the leg supported on the plinth. Each muscle group was tested three times with at least 30 s of rest between trials, and the maximum of the three trials was retained. Values were converted from pounds-force to Newtons using the factor 4.4482216153 N/lbf.

**Figure 2:**
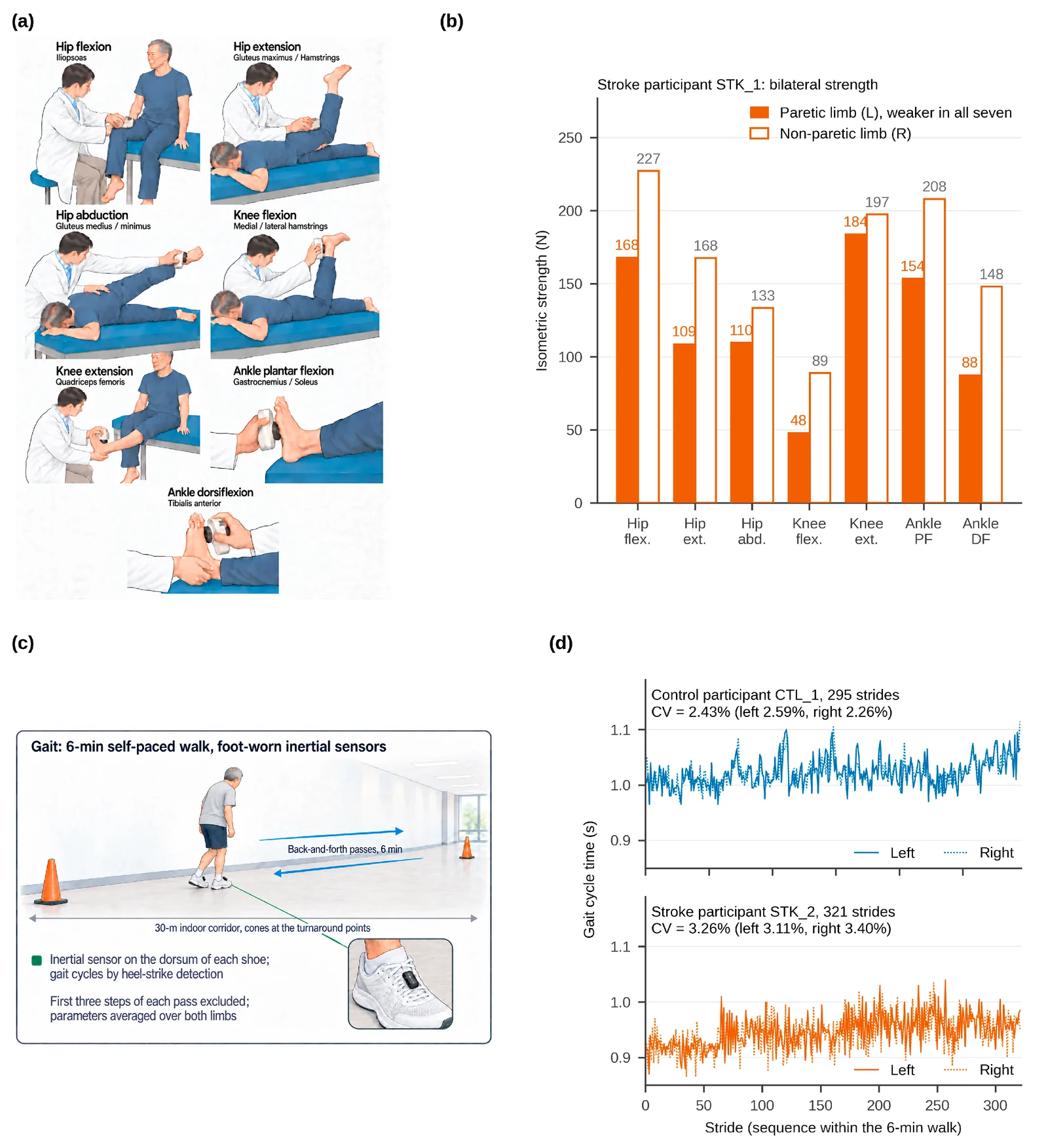
Measurement setup and representative data. (**a**) Handheld dynamometer test positions for the seven muscle groups. (**b**) Bilateral isometric strength (N) of one stroke participant (STK_1, left hemiparesis) for the seven muscle groups; filled bars mark the paretic limb, which was the weaker limb for every muscle group and therefore the value entering the primary analysis. (**c**) Six-minute self-paced walk along a 30-m corridor with foot-worn inertial sensors. (**d**) Stride-by-stride gait cycle time (s) of one control participant (CTL_1) and one stroke participant (STK_2), left and right limbs, with each participant’s coefficient of variation (CV) of gait cycle time as analysed (mean of the two limbs). Participants were selected by rule: in (**d**), the participant closest to the group median CV among participants with stride-level exports; in (**b**), the stroke participant whose weaker-limb values were closest to the stroke-group medians among those with the paretic limb weaker in all seven muscle groups. Participant labels are pseudonyms. flex., flexion; ext., extension; abd., abduction; PF, plantarflexion; DF, dorsiflexion. Labels indicate the principal muscles acting in each tested direction.

The primary strength variable is the smaller of the left and right limb values for each muscle group. This rule was chosen for three reasons: it indexes the more impaired side without requiring the paretic side to be documented, it applies symmetrically to both groups, and it is not defined by the better-preserved limb. The strength block was also analysed with the larger limb, the mean of both limbs, and, in participants with a documented paretic side, the paretic limb (Supplementary Table S13). The dynamometer test configuration and the bilateral values of one stroke participant, illustrating the weaker-limb rule, are shown in Figure 2a,b.

Three left knee-extension values in the stroke group could not be verified against the original measurement records. They were treated as missing, so that the weaker-limb value for knee extension is not defined for those three participants. The strength block was therefore analysed in the 96 participants with complete data, and repeated without knee extension in all 99 participants.

### 2.4. Gait Assessment

Gait was assessed during a self-paced 6-min walk test using two synchronized 7-degree-of-freedom inertial measurement units (Physilog 5; Gait Up SA, Lausanne, Switzerland), each comprising a three-axis accelerometer, three-axis gyroscope, and one-axis barometer, secured to the dorsum of each shoe. The Physilog system has been validated against optical motion capture systems in older adults, stroke survivors, and other clinical populations [21,22,23]. Tests were conducted along a 30-m flat indoor corridor with cones at the turnaround points. The participants completed back-and-forth passes at their preferred walking speed for 6 min (mean 335.79 ± 26.91 gait cycles).

Raw IMU signals were processed using the manufacturer’s software Gait Analysis Toolkit (Gait Up SA, v4.7.0), which identifies individual gait cycles via heel-strike detection and calculates spatiotemporal parameters per stride. The first three steps of each pass were excluded to eliminate acceleration artefacts. Four gait performance variables were retained as means across all valid cycles: gait speed (m/s), cadence (steps/min), gait cycle time (s), and stance phase (%). Swing phase was not analysed as a performance variable because stance and swing sum to 100% by definition, so the two carry the same information. For all participants, each gait parameter was taken as the mean of the two limbs. The sensor placement and walkway, and the stride-by-stride gait cycle times of one control and one stroke participant, are shown in Figure 2c,d. The schematic illustrations in Figure 2a,c were produced with an image-generation tool.

### 2.5. Statistical Analysis

Normality was assessed with the Shapiro–Wilk test and homogeneity of the variance–covariance matrices with Box’s M test. Because several variables violated these assumptions, the outcome variables were rank-transformed before analysis [24].

The primary analysis (Run A) was, for each of three outcome blocks, an additive multivariate analysis of covariance on the ranked outcomes, with stroke status (2 levels) and phenotype group (3 levels: robust, slow gait with preserved muscle mass, low ASM) as factors and sex, age, and height as covariates. The blocks were (1) seven muscle strength variables, (2) three gait performance variables (gait cycle time, cadence, and stance phase), and (3) three gait variability variables (coefficients of variation of gait cycle time, stance phase, and swing phase). The model contained the two main effects and no interaction term, so that each exposure is estimated adjusted for the other and no estimate rests on a single group × phenotype cell.

Four further runs each changed one element of this model. Run B added gait speed as a continuous covariate to the gait performance and gait variability blocks, to estimate the associations at a given walking speed. Run C removed the phenotype, to estimate the stroke association without adjustment for it. Run D added weight, a matching variable, as a covariate. Run E replaced the phenotype with ASM/Ht^2^ as a continuous exposure that does not depend on gait, with gait speed added to the gait performance block as a fourth outcome.

Multivariate effects were tested with Pillai’s trace, and partial eta-squared was computed from its F approximation and degrees of freedom [25]. *p* values were obtained by Freedman–Lane permutation of the residuals of the model without the tested term (9999 permutations) [26], with exchanges restricted to members of the same matched set so that the inference respects the matched design. For each exposure, the block tests of a run formed one family, and *p* values were adjusted with the Holm procedure [27]; decisions in the text are based on the Holm-adjusted *p*. Univariate analyses of covariance on each outcome, with Holm adjustment within each block, are reported as exploratory localisation of significant multivariate effects (Supplementary Table S7).

The stroke × phenotype interaction was examined in a separate exploratory run in which the product terms were added to Run A and tested against it (Supplementary Table S11).

Pairwise phenotype contrasts from Run A (robust versus slow gait, robust versus low ASM, and low ASM versus slow gait) were tested in the same way, with Holm adjustment across all contrasts within a block, as exploratory localisation (Supplementary Table S14). Muscle quality was calculated as weaker-limb knee-extension strength (N) divided by appendicular skeletal muscle mass (kg), because segmental leg lean mass was not available for all participants, and was analysed with the Run A model as a single ranked outcome (n = 96), with Holm adjustment across the three phenotype contrasts (Supplementary Table S15) [28,29].

Sensitivity analyses repeated Run A with the strength block restricted to the six muscle groups other than knee extension (n = 99) and with indicators of the control source cohort added as covariates (Supplementary Table S8), and in each of the five matched datasets constructed during the study (Supplementary Table S9; Supplementary Methods S1). Random-intercept models for matched set are reported in Supplementary Table S10.

All analyses were conducted in Python 3.12 (statsmodels 0.14.6, SciPy 1.15.3, NumPy 2.4.4, pandas 3.0.2; Supplementary Table S16). Significance was set at α = 0.05; exact *p* values are reported, except where <0.001.

## 3. Results

### 3.1. Cohort Characteristics and Covariate Balance

The analysed sample comprised 33 stroke survivors and 66 matched controls in 33 matched sets (Table 1). Matching achieved close balance on all variables entered into the assignment: the standardised mean difference was 0.15 for age, −0.02 for height, and 0.02 for weight, and sex was matched exactly (33.3% women in both groups). Appendicular skeletal muscle mass index (SMD −0.05) and body mass index (SMD 0.05) were also balanced. No absolute standardised mean difference exceeded 0.16. All 66 controls fell within the ±7-year age caliper; the mean absolute age difference within matched sets was 4.6 years (Supplementary Table S4).

**Figure 3:**
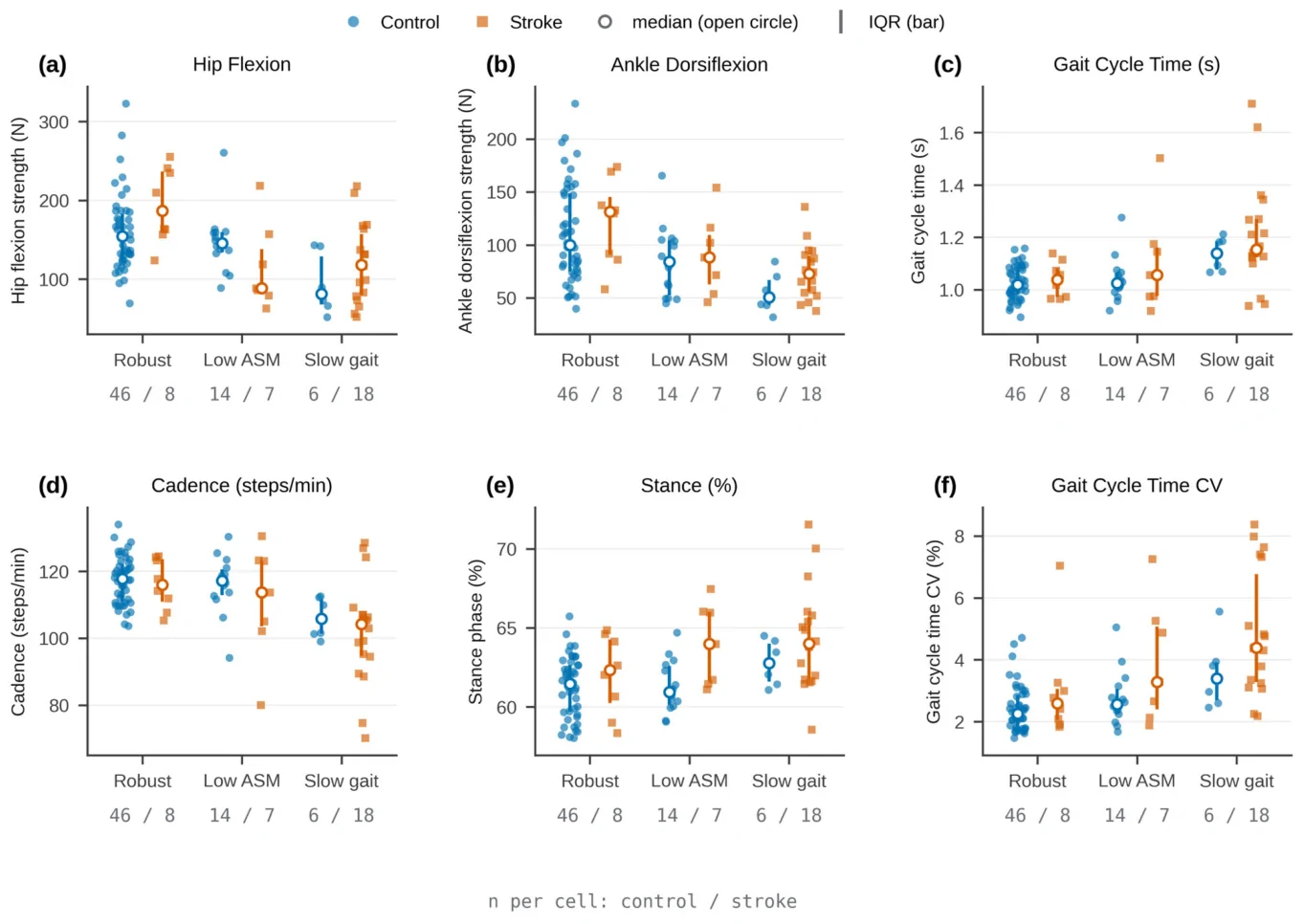
Individual data points with median and interquartile range, by group and phenotype, for six representative outcomes from the three analysed blocks: (**a**) hip flexion strength; (**b**) ankle dorsiflexion strength; (**c**) gait cycle time; (**d**) cadence; (**e**) stance phase; (**f**) gait cycle time coefficient of variation (CV). Each point is one participant (control, circles; stroke, squares), jittered horizontally; the open circle is the cell median and the vertical bar the interquartile range, in raw units. Cell sizes (control/stroke) are given beneath each phenotype.

Phenotype groups were distributed unevenly across the two groups (Table 1). Among stroke survivors, 8 were classified as robust, 18 as slow gait with preserved muscle mass, and 7 as low ASM; among controls the corresponding counts were 46, 6, and 14. Three of the six cells therefore contained fewer than ten participants, and the smallest cell contained six. Descriptive statistics for each group × phenotype cell are given in Supplementary Table S2, and individual values for six representative outcomes are shown in Figure 3.

### 3.2. Primary Analysis

In the primary analysis (Run A; Table 2), stroke was associated with strength (Pillai’s V = 0.240, F(7, 83) = 3.75, *p* = 0.001, Holm *p* = 0.003, partial eta-squared 0.24) and with gait variability (V = 0.096, F(3, 90) = 3.19, *p* = 0.022, Holm *p* = 0.044, 0.10), but not with gait performance (V = 0.064, F(3, 90) = 2.04, *p* = 0.133, Holm *p* = 0.133, 0.06). The phenotype was associated with strength (V = 0.335, F(14, 168) = 2.42, *p* = 0.004, Holm *p* = 0.008, 0.17), gait performance (V = 0.244, F(6, 182) = 4.21, *p* < 0.001, Holm *p* = 0.003, 0.12), and gait variability (V = 0.197, F(6, 182) = 3.31, *p* = 0.006, Holm *p* = 0.008, 0.10).

In the exploratory univariate localisation (Supplementary Table S7), the phenotype effect on strength was present in six of the seven muscle groups after Holm adjustment within the block (all except ankle plantarflexion, Holm *p* = 0.083), whereas no single muscle group carried a stroke effect (smallest Holm *p* = 0.216). In the gait performance block the phenotype effect was present in gait cycle time (F = 10.01, Holm *p* < 0.001) and cadence (F = 9.62, Holm *p* < 0.001). In the gait variability block, stroke effects were present in stance variability (F = 8.34, Holm *p* = 0.011) and swing variability (F = 9.52, Holm *p* = 0.011), and phenotype effects in all three indices (Holm *p* ≤ 0.012).

The pairwise phenotype contrasts placed these differences in the slow-gait group (Supplementary Table S14). After adjustment within each block, participants with low ASM showed no detectable difference from robust participants on any outcome (smallest Holm *p* = 0.381), whereas slow-gait participants were weaker than robust participants in six of the seven muscle groups, with ankle plantarflexion in the same direction (Holm *p* = 0.590), and differed from robust participants in gait cycle time, cadence, and all three variability indices. Participants with low ASM were weaker than robust participants in direction in all seven muscle groups, and their strength did not differ detectably from that of slow-gait participants (Holm *p* ≥ 0.056).

### 3.3. Gait Speed, Removal of the Phenotype, and Weight

With gait speed as a covariate (Run B), neither exposure was associated with gait performance (stroke V = 0.055, Holm *p* = 0.454; phenotype V = 0.105, Holm *p* = 0.247) or gait variability (stroke V = 0.027, Holm *p* = 0.481; phenotype V = 0.037, Holm *p* = 0.805). With the phenotype removed (Run C), stroke was associated with all three blocks: strength (V = 0.347), gait performance (V = 0.194), and gait variability (V = 0.270) (all Holm *p* < 0.001). Read together, Runs A, B, and C place the stroke association with gait performance at V = 0.194 without adjustment for the phenotype, 0.064 with it, and 0.055 at a given gait speed, and the association with gait variability at 0.270, 0.096, and 0.027.

With weight added as a covariate (Run D), the stroke association with strength and the phenotype associations with all three blocks were unchanged in judgement (Holm *p* ≤ 0.008), whereas the stroke association with gait variability was no longer significant after correction (V = 0.087, *p* = 0.034, Holm *p* = 0.068).

### 3.4. Muscle Mass Index in Place of the Phenotype

When the phenotype was replaced by continuous ASM/Ht^2^ (Run E; Supplementary Table S6), ASM/Ht^2^ was not associated with any block (V = 0.036 for strength, 0.009 for gait performance including gait speed, and 0.011 for gait variability; permutation *p* = 0.854–0.879; Holm *p* = 1.000), whereas stroke was associated with all three (V = 0.347, 0.391, and 0.269; all Holm *p* < 0.001).

Muscle quality (weaker-limb knee-extension strength per kilogram of appendicular skeletal muscle mass; n = 96) was associated with the phenotype (*p* < 0.001, partial eta-squared 0.21) but not with stroke (*p* = 0.615, 0.002) (Supplementary Table S15). Slow-gait participants had lower muscle quality than robust participants (Holm *p* = 0.001) and than low-ASM participants (Holm *p* < 0.001), with cell medians of 6.66 and 5.78 N/kg in the stroke and control slow-gait cells against 8.84 to 10.84 N/kg in the other four cells, whereas robust and low-ASM participants did not differ (Holm *p* = 0.132).

### 3.5. Exploratory Interaction

Adding the stroke × phenotype interaction to Run A did not reveal an interaction in any block (Supplementary Table S11): strength V = 0.210, F(14, 164) = 1.38, *p* = 0.126, Holm *p* = 0.378, partial eta-squared 0.105; gait performance V = 0.042, *p* = 0.710, Holm *p* = 1.000, 0.021; gait variability V = 0.030, *p* = 0.727, Holm *p* = 1.000, 0.015.

### 3.6. Sensitivity Analyses

Restricting the strength block to the six muscle groups other than knee extension (n = 99) and adding indicators of the control source cohort changed no judgement of Run A (Supplementary Table S8). The stroke association with strength was significant under all four limb rules (Holm *p* ≤ 0.004), and the phenotype association under the weaker-limb, mean, and paretic-limb rules but not under the stronger-limb rule (*p* = 0.053) (Supplementary Table S13).

Matched datasets. Run A was repeated in each of the four alternative matched datasets (Supplementary Table S9; Supplementary Methods S1). The stroke association with strength was significant in all five datasets (Holm *p* ≤ 0.014 in the alternatives). The other judgements changed in at least one alternative. The stroke association with gait performance, not significant in the primary dataset (Holm *p* = 0.133), was significant in two alternatives (Holm *p* = 0.014 and 0.046); the phenotype association with gait performance was not significant in two (Holm *p* = 0.131 and 0.234); the stroke association with gait variability was not significant in one (Holm *p* = 0.052); and the phenotype associations with strength and gait variability were not significant in one (Holm *p* = 0.056 and 0.234). In one alternative dataset, which has no matched-set identifier, permutations were not restricted within sets.

Random-intercept models for matched set gave intraclass correlations of 0.000 to 0.116 (Supplementary Table S10). Two models did not converge (gait cycle time and cadence), and in two the matched-set variance was estimated at the zero boundary (hip flexion and ankle plantarflexion).

AWGS 2019 concordant classification [14]. When sarcopenia was defined as the conjunction of low ASM and slow gait, only 8 participants were positive (5 stroke, 3 control; Supplementary Table S12). No inferential test was performed on this classification; descriptive counts are reported for reference.

**Figure 4:**
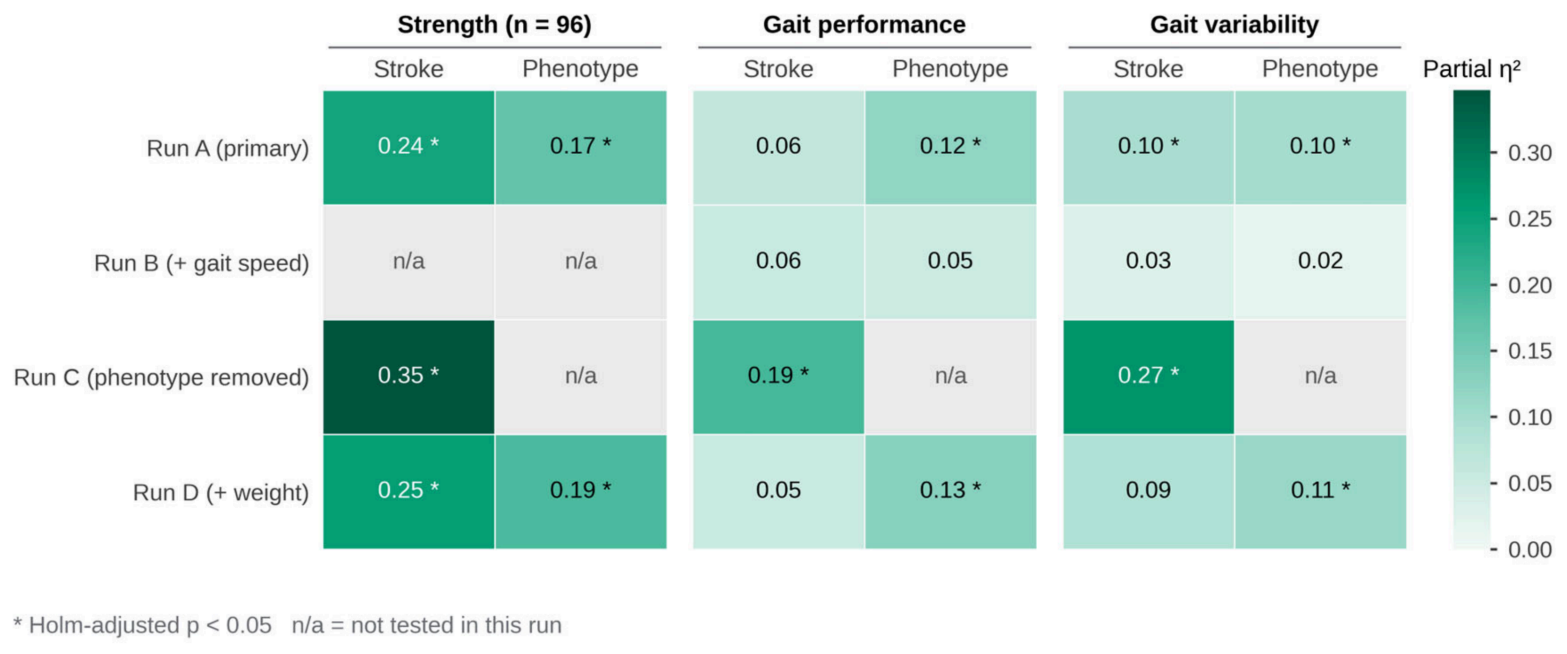
Partial eta-squared of the stroke and phenotype effects in each outcome block across the primary analysis (Run A) and three further runs: gait speed added as a covariate (Run B), phenotype removed (Run C), and weight added (Run D). Asterisks mark Holm-adjusted *p* < 0.05; n/a, not tested in that run. Strength block n = 96; gait blocks n = 99.

The effect sizes of the two exposures across the three blocks in Runs A to D are summarised in Figure 4.

## 4. Discussion

### 4.1. Mutually Adjusted Associations

Stroke and the sarcopenia-related phenotype were each associated with lower-limb function while the other was held constant. Stroke was associated with isometric strength (V = 0.240, Holm *p* = 0.003, partial eta-squared 0.24) and with gait variability (V = 0.096, Holm *p* = 0.044, 0.10). The phenotype was associated with strength (Holm *p* = 0.008, 0.17), gait performance (Holm *p* = 0.003, 0.12), and gait variability (Holm *p* = 0.008, 0.10). These are additive main effects: each is the association of one exposure averaged over the levels of the other, and none depends on the composition of a single group × phenotype cell. In the primary analysis the two exposures therefore overlap on strength and gait variability rather than dividing the outcome space between them.

The weaker-limb rule bears on the stroke association with strength. Both limbs are weaker after stroke than in age-matched controls: Dorsch and colleagues found the non-paretic limb of ambulatory chronic stroke survivors to be significantly weaker than that of controls across muscle groups [30], and a systematic review reported deficits in both paretic and non-paretic limbs relative to age-matched control data [5]. The weaker-limb value of a stroke survivor therefore reflects a bilateral deficit and not hemiparesis on one side alone.

The stroke effect on strength was multivariate rather than localised: no single muscle group carried it after adjustment within the block, while the seven together did. We therefore make no claim about a distal-to-proximal gradient, and we do not identify particular muscle groups as selectively affected.

### 4.2. Gait Speed as One Output of a Multilevel Control System

Walking speed is one output of a control system organised at several levels. Spinal central pattern generators produce the basic locomotor rhythm and the alternation between the limbs [31,32]; supraspinal centres adapt that rhythm to the task and the environment; sensory feedback corrects each cycle; and musculoskeletal capacity bounds what the system can execute. The same speed can be produced by different combinations of step length, cadence, and the timing of the stance and swing phases. This is the redundancy, or abundance, of degrees of freedom described by Bernstein [33] and treated in current motor control as a resource rather than a problem [34]. Stride-to-stride variability, in turn, indexes how consistently the cycle is reproduced rather than its average [35,36]. Stroke alters this organisation at the supraspinal and corticospinal level, whereas low muscle mass limits capacity at the executing end.

Within this frame, the runs answer different questions. Removing the phenotype (Run C) shows how much of the stroke association the phenotype shares. Stroke and the slow-gait level of the phenotype largely coincide in this sample (18 of 33 stroke survivors against 6 of 66 controls), and adjusting for the phenotype reduced the stroke association with gait performance from V = 0.194 to 0.064, with gait variability from 0.270 to 0.096, and with strength from 0.347 to 0.240. The difference is variance the two exposures share, and the analysis cannot assign it to either. The absence of a stroke association with gait performance in the primary analysis is therefore a consequence of adjusting for a classification that contains walking speed, and not evidence that stroke is unrelated to gait performance.

### 4.3. Associations at a Given Walking Speed

Adding gait speed as a covariate (Run B) left no detectable association of either exposure with gait performance or gait variability (stroke Holm *p* = 0.454 and 0.481; phenotype Holm *p* = 0.247 and 0.805). In this cohort the gait associations were therefore not detectable conditional on walking speed: the exposures are associated with how fast participants walked, and the differences in gait timing and variability accompany that difference in speed. For gait cycle time and cadence this follows from their relation to speed, as components from which speed is produced. For stance proportion and variability, which speed does not determine arithmetically, the analysis places the association with the slower walking itself; it does not show whether slow walking produces these differences or whether an impairment of supraspinal or neuromuscular control produces both (Section 4.4).

The stroke association with gait variability was also among the judgements most sensitive to the specification: it did not survive correction for multiplicity when weight was added (Holm *p* = 0.068) or in one alternative matched dataset (Holm *p* = 0.052), although its size changed little (V = 0.087 with weight). It should therefore be read with caution.

### 4.4. Strength and Walking Performance Rather than Muscle Mass

The phenotype contrasts locate the functional differences in one group. Participants with low muscle mass showed no detectable difference from the robust group on any outcome after correction, and ASM/Ht^2^ was not associated with any domain, whereas participants who walked slowly despite preserved muscle mass were weaker in six of the seven muscle groups, with the seventh in the same direction, and differed in gait timing and variability. The differences associated with the phenotype were therefore concentrated in the slow-gait group rather than in the group defined by low muscle quantity, although the low-ASM group was weaker than the robust group in direction. Muscle quality points the same way: strength per kilogram of appendicular muscle mass was lower in the slow-gait group than in the robust group, and because both groups have preserved muscle mass by definition, this is a difference in force within the same range of muscle mass. The low-ASM group had a numerically higher muscle quality than the robust group, which is expected from its smaller denominator, and the difference was not significant. A loss of strength and walking performance with preserved muscle mass corresponds to what has been described as dynapenia [37,38], in which neural factors such as reduced neural drive, motor unit loss, and impaired excitation–contraction coupling contribute more than muscle size [38], and muscle quality has been related to function and to gait variability in older adults [28,29,39]. In stroke survivors the neurological lesion provides such a source directly. Because weakness and slow gait occurred together, these cross-sectional data cannot establish whether weakness slows walking or whether a shared neural or neuromuscular impairment produces both. The lack of a detectable association with muscle mass suggests, however, that muscle quantity is not the main link.

Bioimpedance estimates of appendicular mass carry measurement error, which may have attenuated the association of ASM/Ht^2^, and muscle quality was computed with appendicular rather than leg lean mass. Neither point changes the contrast between the slow-gait and robust groups, which have preserved muscle mass by definition.

### 4.5. Interaction

No interaction between stroke and the phenotype was detected in any block (strength Holm *p* = 0.378, partial eta-squared 0.105; gait performance and gait variability Holm *p* = 1.000). The interaction terms rest on the six group × phenotype cells, three of which contained fewer than ten participants, and the estimates are imprecise, particularly for strength. The absence of a detected interaction is therefore not evidence that the associations are the same in stroke survivors and controls.

### 4.6. Robustness Across Matched Datasets

Five matched datasets were constructed under different matching objectives, and the primary one is the dataset whose objective combined the Mahalanobis distance with the signed age term described in Section 2.1. Only the stroke association with strength held in all five, and it was also present under all four limb rules. The phenotype associations with strength and gait variability and the stroke association with gait variability held in four; the phenotype association with gait performance held in three; and the stroke association with gait performance, absent in the primary dataset, was present in two alternatives. The pattern in which the phenotype but not stroke is associated with gait performance appeared in two of the five datasets. These are the judgements that a different, defensible choice of controls would have changed, and they limit the robustness of every finding other than the stroke association with strength.

The present analysis does not show that the phenotype spares lower-limb strength or that stroke spares gait variability. The claim supported by these data is that both exposures are associated with lower-limb function after mutual adjustment in this cohort, that gait performance is the one domain in which the two exposures reached different judgements in the primary analysis, and that this difference depends on the matched dataset.

### 4.7. Relation to AWGS 2019 and 2025 Criteria

The phenotype used here departs from the AWGS 2019 diagnostic algorithm [14] in two respects: grip strength was not measured, so only the muscle-mass and physical-performance components were applied, and gait speed was derived from a 6-min walk rather than a 6-metre usual-pace test. Applying the AWGS 2019 [14] conjunction of low muscle mass and slow gait to this sample identified only eight participants (five stroke, three control), too few to support any inferential comparison; we report the counts descriptively.

The 2025 AWGS update [40] reframes sarcopenia within a broader concept of muscle health and treats physical performance as a consequence rather than as a diagnostic component. Under that framework the slow-gait group in this study would not be classified as sarcopenic, and the gait findings reported here would be read as describing the relationship between a performance phenotype and gait rather than between sarcopenia and gait. This is a further reason to describe the exposure as a sarcopenia-related phenotype rather than as sarcopenia. The update also places greater weight on muscle strength, the component this study could not include through grip strength, and treats physical performance as an outcome, which is how gait speed is analysed in Run E.

The interpretive boundary that follows should be stated plainly. The phenotype groups are an operational classification built from two of the AWGS 2019 [14] components; they are not a diagnosis of sarcopenia, and no participant in this study was diagnosed with sarcopenia. Membership of the slow-gait group means walking speed below 1.0 m/s with preserved muscle mass index, and membership of the low-ASM group means muscle mass index below the sex-specific cut-off regardless of walking speed. Every association reported for “the phenotype” is an association with that classification. In stroke survivors in particular, slow walking may reflect the neurological lesion rather than a muscle-related process, and the phenotype label does not distinguish between the two.

### 4.8. Limitations

Several limitations qualify these findings. The design was cross-sectional, and the results are conditional associations in a selected cohort; they do not show that stroke and a sarcopenia-related process exert independent biological effects. No formal index-stroke severity measure was available, and clinical characterisation was missing for three stroke participants. The controls were drawn from five institutional cohorts rather than a single sampling frame; adding source-cohort indicators changed no judgement, but differences in measurement setting cannot be fully excluded. Physical activity, comorbidity burden, and medication were not recorded in a common form across the source cohorts and were unavailable for most participants, so these potential confounders could not be adjusted for. Grip strength was not available, so the phenotype could not be defined by the full AWGS 2019 algorithm [14]. Three of the six group × phenotype cells contained fewer than ten participants, which limits the precision of the interaction analysis. The strength block was analysed in 96 participants because three knee-extension values could not be verified. Neural measures such as electromyography, motor unit properties, or cognition were not recorded, so the neuromuscular interpretation of the phenotype findings remains an inference. Muscle quality was computed with appendicular rather than leg lean mass, because segmental lean mass was not available for all controls. Finally, several judgements depended on the matched dataset (Section 4.6).

### 4.9. Clinical Considerations

Randomised trials in stroke survivors have produced mixed results. Twelve weeks of eccentric-overload flywheel resistance training of the more-affected lower limb increased quadriceps volume by 9.4% and muscle power by 48.2% in the trained limb, with concurrent gains in gait performance [41]. Nutritional interventions have been less consistent: whey protein with vitamin D over 16 weeks reduced intramuscular fat infiltration but did not change muscle cross-sectional area, skeletal muscle index, or strength [42], and creatine supplementation during the first week after acute stroke did not affect functional capacity, strength, or muscle mass at 7 or 90 days [43]. The present cross-sectional study does not test any intervention. It indicates that, in this cohort, lower strength accompanied both a history of stroke and a slow-gait presentation, and that the gait associations of both were not detectable conditional on walking speed.

## 5. Conclusions

In this 1:2 matched cohort of 99 community-dwelling older adults, lower-limb function followed strength and walking performance more closely than muscle mass. Stroke and an operationally defined sarcopenia-related phenotype (a classification, not a diagnosis of sarcopenia) were each associated with lower-limb function after mutual adjustment. The stroke association with strength held in every matched dataset and under every limb rule, whereas its association with gait variability was not significant after adjustment for weight. The phenotype’s associations with the three domains held in the primary matched dataset and in three or four of the five matched datasets; in the primary dataset they were carried by participants who walked slowly despite preserved muscle mass, who were weaker and had lower muscle quality, while low muscle mass showed no detectable association with any outcome. Neither exposure’s gait association was detectable conditional on walking speed. No interaction was detected, and the estimates are imprecise, particularly for strength. These are conditional cross-sectional associations in a selected cohort.

## Figures and Tables

**Table 1:** Participant characteristics and covariate balance (n = 99).

Variable	Stroke (n = 33)	Control (n = 66)	SMD	Variance Ratio	*p*
Age (y)	72.42 ± 7.21	73.45 ± 6.22	0.153	0.743	0.486
Height (cm)	163.12 ± 9.13	162.97 ± 8.43	−0.017	0.852	0.937
Weight (kg)	64.78 ± 9.72	64.92 ± 9.16	0.015	0.888	0.946
BMI (kg/m^2^)	24.30 ± 2.80	24.45 ± 3.00	0.051	1.148	0.810
ASM/Ht^2^ (kg/m^2^)	7.19 ± 1.05	7.15 ± 0.85	−0.048	0.667	0.829
Female, n (%)	11 (33.3)	22 (33.3)	0.000		1.000
Phenotype: Robust, n	8	46			
Phenotype: Slow gait, preserved mass, n	18	6			
Phenotype: Low ASM, n	7	14			

Values are mean ± SD or n (%). SMD, standardised mean difference (control minus stroke). *p* values are shown for reference and were not used to judge balance.

**Table 2:** Multivariate associations of stroke and phenotype with the three outcome blocks in the primary analysis and three further runs.

Run	Strength		Gait Performance		Gait Variability	
	Stroke	Phenotype	Stroke	Phenotype	Stroke	Phenotype
A (primary)	0.240 0.001 0.003 0.24	0.335 0.004 0.008 0.17	0.064 0.133 0.133 0.06	0.244 <0.001 0.003 0.12	0.096 0.022 0.044 0.10	0.197 0.006 0.008 0.10
B (+gait speed)	n/a	n/a	0.055 0.227 0.454 0.06	0.105 0.124 0.247 0.05	0.027 0.481 0.481 0.03	0.037 0.805 0.805 0.02
C (phenotype removed)	0.347 <0.001 <0.001 0.35	n/a	0.194 <0.001 <0.001 0.19	n/a	0.270 <0.001 <0.001 0.27	n/a
D (+weight)	0.255 <0.001 0.002 0.25	0.377 0.003 0.007 0.19	0.053 0.208 0.208 0.05	0.261 0.002 0.005 0.13	0.087 0.034 0.068 0.09	0.225 0.008 0.008 0.11

Each cell gives, from top to bottom, Pillai’s V, the permutation *p* value, the Holm-adjusted *p* value, and partial eta-squared. Run A: stroke + phenotype, adjusted for sex, age, and height. Run B: Run A + gait speed as a covariate (gait blocks only). Run C: Run A without the phenotype. Run D: Run A + weight. Outcomes rank-transformed. *p* values by Freedman–Lane permutation (9999 permutations) restricted within matched sets; Holm adjustment across the blocks of a run for each exposure. Strength block n = 96 (complete case); gait blocks n = 99. n/a, not part of the run. Full statistics are given in Supplementary Table S5.

## Data Availability

The data and analysis code presented in this study are available on request from the corresponding author due to privacy and ethical restrictions. The data are not publicly available because they contain individual-level clinical information from a small clinical cohort, and public release is not covered by the institutional ethics approval or by participants’ informed consent.

## Supplementary Materials

The following supporting information can be downloaded at: https://www.mdpi.com/article/10.3390/jcm15207779/s1, Supplementary Methods S1. Matching specification: objective function, constraints, solver, and the five matched datasets. Table S1. Clinical characteristics of the stroke group (n = 33). Table S2. Descriptive statistics by group × phenotype cell. Table S3. Source cohorts of the matched controls. Table S4. Age caliper distribution within matched sets. Table S5. Multivariate results of Runs A–D. Table S6. Run E: continuous ASM/Ht^2^ in place of the phenotype (gait performance block with gait speed as a fourth outcome). Table S7. Univariate analyses of covariance for Run A (exploratory localisation of significant multivariate effects). Table S8. Sensitivity runs: strength block without knee extension (n = 99) and source-cohort indicators added. Table S9. Run A in the five matched datasets. Table S10. Random-intercept models for matched set: intraclass correlations. Table S11. Exploratory interaction run. Table S12. AWGS 2019 concordant classification. Table S13. Limb rules: strength block under the weaker-limb, stronger-limb, mean, and paretic-limb rules. Table S14. Pairwise phenotype contrasts for every outcome (exploratory localisation). Table S15. Muscle quality: weaker-limb knee-extension strength per kilogram of appendicular skeletal muscle mass. Table S16. Software environment and data dictionary.
