# Supplementary material for: Independent Associations of Stroke and Sarcopenia-Related Phenotype with Lower-Limb Strength and Gait: A 1:2 Matched Cohort Analysis
Source: J Clin Med. 2026 Oct 9;15(20):7779. doi: 10.3390/jcm15207779 (PMC13705296; doi:10.3390/jcm15207779)
Supplement: Supplementary file 1 [file jcm-15-07779-s001.zip › jcm-4582702-supplementary.pdf]

Independent Associations of Stroke and Sarcopenia-Related Phenotype with Lower-Limb Strength and Gait: A 1:2 Matched Cohort Analysis

Supplementary Materials

Supplementary Methods S1. Matching specification: objective function, constraints, solver, and the five matched datasets

S1.1 Eligible pool. The eligible control pool comprised the 259 individuals remaining after the exclusions and the person-level deduplication described in Section 2.1 and Figure 1.

S1.2 Distance. For stroke case i and candidate control j, with x = (age, height, weight),

$$D(i, j) = \sqrt{[(x_i - x_j)^T S^{-1} (x_i - x_j)]},$$

where S is the sample covariance matrix (divisor n − 1) of x over the 259 eligible controls. Stroke cases do not contribute to S. Sex is not part of x because it is matched exactly (S1.3).

S1.3 Constraints. Assigning control j to case i is feasible only if $sex_i = sex_j$ and $|age_i - age_j| \leq 7$ years. Infeasible pairs receive a prohibitive cost of 10^{15} and are never selected.

S1.4 Cost. For a feasible pair,

$$c(i, j) = D(i, j) - \mu\,(age_j - age_i),\quad \mu = 10^6.$$

The second term is a signed age term: it lowers the cost of older controls. Ages are recorded in whole years, and the largest possible total distance over 66 assignments (66 × max D = 336.8) is far smaller than μ. The objective is therefore lexicographic: among feasible assignments, the total age of the selected controls is maximised first, and the total Mahalanobis distance is minimised second. The term was included because the eligible pool was younger than the stroke cases, so that minimising distance alone produces a control group younger than the cases.

S1.5 Solver. Each case is represented by two slots (66 slots). A one-to-one assignment of slots to distinct controls (without replacement, at the level of the individual) that minimises the total cost $\Sigma\, c(i(s), \pi(s))$ is obtained with scipy.optimize.linear_sum_assignment on the 66 × 259 cost matrix; a constant shift of the matrix, applied for numerical convenience, does not change the minimiser because every solution uses exactly 66 cells. The procedure is deterministic and uses no random numbers. The two controls assigned to the slots of a case form that case's matched set. The solution has a total Mahalanobis distance of 66.64 and a mean absolute within-set age difference of 4.6 years (maximum 7; Supplementary Table S4).

S1.6 Status of the specification. The specification was not registered in a dated protocol before the outcomes were examined. For this reason the results are reported for all five matched datasets constructed during the study (S1.7; Supplementary Table S9).

S1.7 The five matched datasets.

A1 (primary): as specified in S1.1–S1.5.

A2: the same constraints and solver, with the cost equal to the Mahalanobis distance alone (no signed age term) and the covariance computed over the 262-person pool before the removal of the two cross-cohort duplicates and the one unresolved pair.

A3: a matched dataset in which controls were allowed to serve more than one case (66 control rows correspond to 50 individuals) and no matched-set identifier was retained, so in this dataset the permutations were not restricted within matched sets.

A4: Mahalanobis distance on age, sex, height, and weight with the covariance of the 262-person pool; sex matched exactly; a tiered age caliper (5 years preferred, 7 years maximum, with two cases allowed up to 10 years); no signed age term; random seed 20260816.

A5: greedy nearest-neighbour matching of cases in descending order of age, using the Mahalanobis distance on age, sex, height, and weight with the covariance of an augmented 220-person pool; an age caliper of 5 years relaxed to 7 years; 50 controls carried over from an earlier assignment; one case retained a single control (n = 98); random seed 20260816.

Table S1. Clinical characteristics of the stroke group (n = 33)

Variable	n available	n missing	Summary
time since stroke (mo)	30	3	106.73 ± 78.04
stroke type	30	3	Ischaemic 24, hemorrhagic 6
hemiparetic side (text)	30	3	L 20, R 10
Hemiparetic_side (code)	30	3	0.0 20, 1.0 10
walking aid	30	3	no 22, yes 8
MMSE	29	4	24.21 ± 4.14
RMI	30	3	11.97 ± 1.92
TUG	30	3	15.64 ± 6.53
education (y)	30	3	16.10 ± 5.84
falls (prev. year)	30	3	0.63 ± 1.35

Clinical intake records were available for 30 of the 33 stroke participants. The three participants without a clinical record are counted as missing on every clinical variable.

Table S2. Descriptive statistics by group × phenotype cell

group	phenotype	n	age	Hip Flexion	Hip Extension	Hip Abduction	Knee Flexion	Knee Extension	Ankle Plantarflexion	Ankle Dorsiflexion	Gait Cycle Time (s)	Cadence (steps/min)	Stance (%)	Gait Speed (m/s)	Gait Cycle Time CV	Stance CV	Swing CV
Stroke	Robust	8	69.4 ± 9.3	193.679	149.322	115.760	83.026	219.187	171.122	122.484	1.038	116.148	62.041	1.249	3.041	3.018	4.845
Stroke	Low ASM	7	75.0 ± 5.9	116.083	100.271	89.984	66.932	155.582	105.923	90.428	1.107	111.149	63.968	0.845	3.909	3.243	5.805
Stroke	Slow gait	18	72.8 ± 6.6	120.570	97.798	81.892	53.517	127.221	122.127	74.608	1.210	101.902	64.366	0.752	4.863	3.755	6.957
Control	Robust	46	72.1 ± 6.3	159.314	120.614	116.979	78.569	182.667	176.875	111.264	1.026	117.419	61.289	1.295	2.430	2.217	3.470
Control	Low ASM	14	75.7 ± 4.7	146.982	89.822	97.003	55.380	176.499	183.998	84.421	1.039	116.213	61.314	1.237	2.764	2.240	3.561
Control	Slow gait	6	78.5 ± 5.6	94.376	64.647	62.868	45.520	121.066	91.040	55.232	1.135	106.129	62.788	0.879	3.559	2.854	4.837

Values are cell means in raw units; age is mean ± SD.

Knee extension was treated as missing for three stroke participants whose left-limb value could not be verified; the knee-extension means of the affected cells are based on n = 7 (stroke, robust) and n = 16 (stroke, slow gait).

Table S3. Source cohorts of the matched controls

Source	Stroke, n	Control, n	Share of controls, %
Stroke group	33	0	–
Cohort 1 (reference)	0	41	62.1
Cohort 2	0	12	18.2
Cohort 3	0	5	7.6
Cohort 4	0	5	7.6
Cohort 5	0	3	4.5
**Total**	33	66	100.0

Controls came from five institutional cohorts, labelled Cohort 1 to Cohort 5 in descending order of the number of controls; institutional cohort codes are not reported. Source-cohort indicators used in the sensitivity analysis (Supplementary Table S8): Cohort 1 is the reference; for each of Cohorts 2 to 5, D_k = 1{source = Cohort k} − p_k for controls and 0 for stroke cases, where p_k is the share of the 66 controls from Cohort k (Cohort 2, 0.181818; Cohort 3, 0.075758; Cohort 4, 0.075758; Cohort 5, 0.045455). The indicators are centred within the controls, so the stroke coefficient compares stroke cases with the average control mix.

Table S4. Age caliper distribution within matched sets

|Δ age| (years)	Pairs	%
0 ≤ |Δage| < 1	1	1.5
1 ≤ |Δage| < 2	6	9.1
2 ≤ |Δage| < 3	8	12.1
3 ≤ |Δage| < 4	11	16.7
4 ≤ |Δage| < 5	3	4.5
5 ≤ |Δage| < 6	8	12.1
6 ≤ |Δage| < 7	8	12.1
7 ≤ |Δage| < 8	21	31.8
> 7 (caliper violation)	0	0.0

Case–control pairs, n = 66. Mean absolute age difference 4.58 years; maximum 7 years. No pair exceeded the ±7-year caliper.

Table S5. Multivariate results of Runs A–D

Block	Tested term	n	Pillai's V	F	df1	df2	Parametric p	Permutation p	Holm p	ηp²
Run A: stroke + phenotype + sex, age, height (main analysis)										
Strength	Stroke	96	0.240	3.75	7	83	0.001	0.001	0.003	0.240
Strength	Phenotype	96	0.335	2.42	14	168	0.004	0.004	0.008	0.168
Gait performance	Stroke	99	0.064	2.04	3	90	0.114	0.133	0.133	0.064
Gait performance	Phenotype	99	0.244	4.21	6	182	<0.001	<0.001	0.003	0.122
Gait variability	Stroke	99	0.096	3.19	3	90	0.027	0.022	0.044	0.096
Gait variability	Phenotype	99	0.197	3.31	6	182	0.004	0.006	0.008	0.098
Run B: Run A + gait speed (continuous covariate); gait blocks only										
Gait performance	Stroke	99	0.055	1.74	3	89	0.165	0.227	0.454	0.055
Gait performance	Phenotype	99	0.105	1.66	6	180	0.133	0.124	0.247	0.052
Gait variability	Stroke	99	0.027	0.83	3	89	0.482	0.481	0.481	0.027
Gait variability	Phenotype	99	0.037	0.57	6	180	0.752	0.805	0.805	0.019
Run C: Run A without the phenotype										
Strength	Stroke	96	0.347	6.46	7	85	<0.001	<0.001	<0.001	0.347
Gait performance	Stroke	99	0.194	7.37	3	92	<0.001	<0.001	<0.001	0.194
Gait variability	Stroke	99	0.270	11.32	3	92	<0.001	<0.001	<0.001	0.270
Run D: Run A + weight										
Strength	Stroke	96	0.255	4.01	7	82	<0.001	<0.001	0.002	0.255
Strength	Phenotype	96	0.377	2.76	14	166	0.001	0.003	0.007	0.189
Gait performance	Stroke	99	0.053	1.67	3	89	0.180	0.208	0.208	0.053
Gait performance	Phenotype	99	0.261	4.50	6	180	<0.001	0.002	0.005	0.131
Gait variability	Stroke	99	0.087	2.83	3	89	0.043	0.034	0.068	0.087
Gait variability	Phenotype	99	0.225	3.80	6	180	0.001	0.008	0.008	0.112

p values by Freedman–Lane permutation (9999 permutations) restricted within matched sets; Holm adjustment across the three blocks (two in Run B) for each exposure within a run. Strength block n = 96 (complete case).

Run B was not applied to the strength block. Parametric p, F approximation of Pillai's trace; ηp², partial eta-squared = F·df1 / (F·df1 + df2).

Table S6. Run E: continuous ASM/Ht² in place of the phenotype (gait performance block with gait speed as a fourth outcome)

Block	Tested term	n	Pillai's V	F	df1	df2	Parametric p	Permutation p	Holm p	ηp²
Run E: stroke + ASM/Ht² (continuous) + sex, age, height										
Strength	Stroke	96	0.347	6.39	7	84	<0.001	<0.001	<0.001	0.347
Strength	ASM/Ht²	96	0.036	0.44	7	84	0.871	0.854	1.000	0.036
Gait performance (4 incl. gait speed)	Stroke	99	0.391	14.46	4	90	<0.001	<0.001	<0.001	0.391
Gait performance (4 incl. gait speed)	ASM/Ht²	99	0.009	0.20	4	90	0.935	0.858	1.000	0.009
Gait variability	Stroke	99	0.269	11.17	3	91	<0.001	<0.001	<0.001	0.269
Gait variability	ASM/Ht²	99	0.011	0.35	3	91	0.791	0.879	1.000	0.011

ASM/Ht² entered as a continuous covariate in place of the phenotype; the gait performance block comprises gait cycle time, cadence, stance phase, and gait speed. Permutation p and Holm adjustment as in Supplementary Table S5 (three blocks per exposure). Strength block n = 96 (complete case).

Table S7. Univariate analyses of covariance for Run A (exploratory localisation of significant multivariate effects)

Outcome	n	Stroke				Phenotype			
		F (df1, df2)	Permutation p	Holm p	ηp²	F (df1, df2)	Permutation p	Holm p	ηp²
Strength									
Hip flexion	96	0.16 (1, 89)	0.621	1.000	0.002	8.98 (2, 89)	<0.001	0.004	0.168
Hip extension	96	3.30 (1, 89)	0.031	0.216	0.036	5.48 (2, 89)	0.005	0.011	0.110
Hip abduction	96	0.17 (1, 89)	0.632	1.000	0.002	11.79 (2, 89)	<0.001	0.001	0.209
Knee flexion	96	1.94 (1, 89)	0.081	0.487	0.021	6.41 (2, 89)	<0.001	0.004	0.126
Knee extension	96	0.36 (1, 89)	0.478	1.000	0.004	8.02 (2, 89)	0.002	0.005	0.153
Ankle plantarflexion	96	3.78 (1, 89)	0.092	0.487	0.041	4.54 (2, 89)	0.083	0.083	0.092
Ankle dorsiflexion	96	0.97 (1, 89)	0.262	1.000	0.011	9.42 (2, 89)	<0.001	0.002	0.175
Gait performance									
Gait cycle time	99	0.22 (1, 92)	0.643	1.000	0.002	10.01 (2, 92)	<0.001	<0.001	0.179
Cadence	99	0.21 (1, 92)	0.645	1.000	0.002	9.62 (2, 92)	<0.001	<0.001	0.173
Stance phase	99	6.19 (1, 92)	0.007	0.022	0.063	1.99 (2, 92)	0.140	0.140	0.041
Gait variability									
Gait cycle time CV	99	4.29 (1, 92)	0.061	0.061	0.045	8.95 (2, 92)	0.003	0.007	0.163
Stance phase CV	99	8.34 (1, 92)	0.005	0.011	0.083	3.44 (2, 92)	0.012	0.012	0.070
Swing phase CV	99	9.52 (1, 92)	0.004	0.011	0.094	5.27 (2, 92)	0.002	0.007	0.103

Run A model (sex, age, height, stroke, phenotype) fitted to each rank-transformed outcome; F test of each exposure against the model without it. p values by Freedman–Lane permutation (9999 permutations) restricted within matched sets; Holm adjustment within each block for each exposure. Exploratory localisation of significant multivariate effects; ηp², partial eta-squared.

Table S8. Sensitivity runs: strength block without knee extension (n = 99) and source-cohort indicators added

Block	Tested term	n	Pillai's V	F	df1	df2	Parametric p	Permutation p	Holm p	ηp²
Strength block without knee extension (six muscles), n = 99										
Strength (6 muscles)	Stroke	99	0.213	3.93	6	87	0.002	0.002	0.004	0.213
Strength (6 muscles)	Phenotype	99	0.298	2.57	12	176	0.004	0.004	0.008	0.149
Run A + source-cohort indicators										
Strength	Stroke	96	0.228	3.33	7	79	0.004	0.002	0.006	0.228

Block	Tested term	n	Pillai's V	F	df1	df2	Parametric p	Permutation p	Holm p	ηp²
Strength	Phenotype	96	0.295	1.98	14	160	0.023	0.012	0.014	0.148
Gait performance	Stroke	99	0.073	2.27	3	86	0.086	0.103	0.103	0.073
Gait performance	Phenotype	99	0.235	3.87	6	174	0.001	0.003	0.008	0.118
Gait variability	Stroke	99	0.118	3.84	3	86	0.012	0.011	0.022	0.118
Gait variability	Phenotype	99	0.194	3.12	6	174	0.006	0.007	0.014	0.097

Strength block without knee extension: the six remaining muscle groups in all 99 participants; its Holm family is the Run A family with the strength test replaced by the six-muscle test. Source-cohort indicators as defined in Supplementary Table S3, added to the Run A covariates; Holm adjustment across the three blocks for each exposure. p values by Freedman–Lane permutation (9999 permutations) restricted within matched sets. No Holm-adjusted judgement at α = 0.05 differs from Run A (a difference would be marked †).

Table S9. Run A in the five matched datasets

Block	Tested term	n	Pillai's V	F (df1, df2)	Permutation p	Holm p	ηp²
Dataset A1 (primary)							
Strength	Stroke	96	0.240	3.75 (7, 83)	0.001	0.003	0.240
Strength	Phenotype	96	0.335	2.42 (14, 168)	0.004	0.008	0.168
Gait performance	Stroke	99	0.064	2.04 (3, 90)	0.133	0.133	0.064
Gait performance	Phenotype	99	0.244	4.21 (6, 182)	<0.001	0.003	0.122
Gait variability	Stroke	99	0.096	3.19 (3, 90)	0.022	0.044	0.096
Gait variability	Phenotype	99	0.197	3.31 (6, 182)	0.006	0.008	0.098
Dataset A2							
Strength	Stroke	96	0.188	2.75 (7, 83)	0.007	0.014	0.188
Strength	Phenotype	96	0.408	3.08 (14, 168)	<0.001	0.002	0.204
Gait performance	Stroke	99	0.120	4.09 (3, 90)	0.013	0.014†	0.120
Gait performance	Phenotype	99	0.110	1.76 (6, 182)	0.131	0.131†	0.055
Gait variability	Stroke	99	0.170	6.15 (3, 90)	<0.001	0.001	0.170
Gait variability	Phenotype	99	0.146	2.38 (6, 182)	0.017	0.035	0.073
Dataset A3							
Strength	Stroke	96	0.229	3.53 (7, 83)	0.002	0.004	0.229
Strength	Phenotype	96	0.328	2.35 (14, 168)	0.005	0.014	0.164
Gait performance	Stroke	99	0.085	2.78 (3, 90)	0.046	0.046†	0.085
Gait performance	Phenotype	99	0.161	2.66 (6, 182)	0.017	0.017	0.081
Gait variability	Stroke	99	0.176	6.41 (3, 90)	<0.001	0.002	0.176
Gait variability	Phenotype	99	0.187	3.12 (6, 182)	0.006	0.014	0.093
Dataset A4							
Strength	Stroke	96	0.249	3.93 (7, 83)	0.001	0.004	0.249
Strength	Phenotype	96	0.316	2.25 (14, 168)	0.010	0.027	0.158
Gait performance	Stroke	99	0.071	2.30 (3, 90)	0.086	0.086	0.071
Gait performance	Phenotype	99	0.155	2.55 (6, 182)	0.042	0.042	0.077
Gait variability	Stroke	99	0.116	3.92 (3, 90)	0.026	0.052†	0.116
Gait variability	Phenotype	99	0.179	2.98 (6, 182)	0.009	0.027	0.089
Dataset A5							
Strength	Stroke	95	0.211	3.14 (7, 82)	0.003	0.009	0.211
Strength	Phenotype	95	0.278	1.92 (14, 166)	0.019	0.056†	0.139
Gait performance	Stroke	98	0.056	1.76 (3, 89)	0.223	0.223	0.056
Gait performance	Phenotype	98	0.156	2.54 (6, 180)	0.151	0.234†	0.078
Gait variability	Stroke	98	0.138	4.74 (3, 89)	0.004	0.009	0.138
Gait variability	Phenotype	98	0.161	2.63 (6, 180)	0.117	0.234†	0.081

Run A refitted in each of the five matched datasets defined in Supplementary Methods S1.7; p values by Freedman–Lane permutation (9999 permutations) restricted within matched sets; Holm adjustment across the three blocks for each exposure within a dataset. Dataset A3 has no matched-set identifier, so its p values come from unrestricted permutation. Dataset A5 comprises n = 98 participants (strength block n = 95) because one case retained a single control. † Holm-adjusted judgement at α = 0.05 differs from dataset A1.

Table S10. Random-intercept models for matched set: intraclass correlations

Outcome	n	Matched sets	σ² set	σ² residual	ICC	Zero boundary	Convergence
Strength							
Hip flexion	96	33	0.0	414.5	0.000	Yes	Converged
Hip extension	96	33	49.0	499.1	0.089	No	Converged
Hip abduction	96	33	8.0	351.9	0.022	No	Converged
Knee flexion	96	33	46.5	510.4	0.083	No	Converged
Knee extension	96	33	29.1	442.5	0.062	No	Converged
Ankle plantarflexion	96	33	0.0	421.9	0.000	Yes	Converged
Ankle dorsiflexion	96	33	16.2	513.4	0.031	No	Converged
Gait performance							
Gait cycle time	99	33	2.8	562.5	0.005	No	Not converged
Cadence	99	33	3.0	567.2	0.005	No	Not converged
Stance phase	99	33	53.5	589.3	0.083	No	Converged
Gait variability							
Gait cycle time CV	99	33	0.5	526.9	0.001	No	Converged after optimizer retry
Stance phase CV	99	33	52.9	534.0	0.090	No	Converged
Swing phase CV	99	33	63.3	481.4	0.116	No	Converged

Random intercept for matched set with the fixed effects of Run A, each outcome rank-transformed (variances on the rank scale), maximum likelihood; ICC = σ² set / (σ² set + σ² residual). Zero boundary: the set variance was estimated at the zero boundary (boundary warning or σ² set below 10^{-6}); 2 of the 13 outcomes. 2 models ended without convergence and 1 converged after an optimizer retry; the ICC is shown as estimated. Strength block n = 96 (complete case).

Table S11. Exploratory interaction run

Block	n	Pillai's V	F (df1, df2)	Permutation p	Holm p	ηp²
Strength	96	0.210	1.38 (14, 164)	0.126	0.378	0.105
Gait performance	99	0.042	0.64 (6, 178)	0.710	1.000	0.021
Gait variability	99	0.030	0.45 (6, 178)	0.727	1.000	0.015

Exploratory run: Run A plus the two stroke × phenotype product terms, tested against Run A. p values by Freedman–Lane permutation (9999 permutations) restricted within matched sets; Holm adjustment across the three blocks. Strength block n = 96 (complete case).

Table S12. AWGS 2019 concordant classification

Group	Low ASM	Slow gait	n
control	N	N	46
control	N	Y	6
control	Y	N	11
control	Y	Y	3
stroke	N	N	8
stroke	N	Y	18
stroke	Y	N	2
stroke	Y	Y	5

Descriptive counts only; no inferential test was performed. The conjunction of low muscle mass and slow gait identifies 8 participants (5 stroke, 3 control).

Table S13. Limb rules: strength block under the weaker-limb, stronger-limb, mean, and paretic-limb rules

Rule	n	Muscles	Tested term	Pillai's V	F	df1	df2	Permutation p	Holm p	ηp²
Weaker limb (primary)	96	7	Stroke	0.240	3.75	7	83	0.001	0.003	0.240
Weaker limb (primary)	96	7	Phenotype	0.335	2.42	14	168	0.004	0.008	0.168
Stronger limb	96	7	Stroke	0.245	3.84	7	83	0.001	0.004	0.245
Stronger limb	96	7	Phenotype	0.296	2.08	14	168	0.053	0.053	0.148
Mean of both limbs	96	7	Stroke	0.244	3.83	7	83	<0.001	0.002	0.244
Mean of both limbs	96	7	Phenotype	0.324	2.32	14	168	0.011	0.011	0.162
Paretic limb	90	6	Stroke	0.288	5.26	6	78	<0.001	<0.001	0.288
Paretic limb	90	6	Phenotype	0.263	1.99	12	158	0.016	0.016	0.132

Strength block, Run A model (stroke + phenotype + sex, age, height) on ranks; p values by Freedman–Lane permutation (9999 permutations) restricted within matched sets. Holm p: the rule's strength test substituted for the strength test in Run A's family of three blocks for that exposure (as in Supplementary Table S8). Weaker-limb, stronger-limb and mean rules: knee extension complete case (n = 96). Paretic-limb rule: stroke participants with a documented paretic side and their matched sets (30 sets, n = 90), six muscles (knee extension unavailable); controls contribute the mean of both limbs, as in the paretic-limb rule definition.

Table S14. Pairwise phenotype contrasts for every outcome (exploratory localisation)

Outcome	Contrast	Direction	F	Permutation p	Holm p	ηp²
Strength						
Hip flexion	Robust vs slow gait	Robust > Slow gait	17.46	<0.001	0.008	0.164
Hip flexion	Robust vs low ASM	Robust > Low ASM	5.05	0.094	0.590	0.054
Hip flexion	Low ASM vs slow gait	Low ASM > Slow gait	4.05	0.024	0.259	0.044
Hip extension	Robust vs slow gait	Robust > Slow gait	9.41	0.002	0.034	0.096
Hip extension	Robust vs low ASM	Robust > Low ASM	5.39	0.074	0.590	0.057
Hip extension	Low ASM vs slow gait	Low ASM > Slow gait	0.82	0.209	0.626	0.009
Hip abduction	Robust vs slow gait	Robust > Slow gait	23.50	<0.001	0.004	0.209
Hip abduction	Robust vs low ASM	Robust > Low ASM	4.51	0.038	0.381	0.048
Hip abduction	Low ASM vs slow gait	Low ASM > Slow gait	7.56	0.007	0.094	0.078
Knee flexion	Robust vs slow gait	Robust > Slow gait	12.54	<0.001	0.008	0.123
Knee flexion	Robust vs low ASM	Robust > Low ASM	3.42	0.124	0.590	0.037
Knee flexion	Low ASM vs slow gait	Low ASM > Slow gait	3.08	0.017	0.216	0.033
Knee extension	Robust vs slow gait	Robust > Slow gait	15.62	0.002	0.035	0.149
Knee extension	Robust vs low ASM	Robust > Low ASM	0.83	0.378	0.755	0.009
Knee extension	Low ASM vs slow gait	Low ASM > Slow gait	8.64	0.019	0.232	0.089
Ankle plantarflexion	Robust vs slow gait	Robust > Slow gait	8.60	0.074	0.590	0.088
Ankle plantarflexion	Robust vs low ASM	Robust > Low ASM	0.24	0.744	0.755	0.003
Ankle plantarflexion	Low ASM vs slow gait	Low ASM > Slow gait	5.50	0.051	0.462	0.058
Ankle dorsiflexion	Robust vs slow gait	Robust > Slow gait	18.57	<0.001	0.004	0.173
Ankle dorsiflexion	Robust vs low ASM	Robust > Low ASM	4.52	0.104	0.590	0.048
Ankle dorsiflexion	Low ASM vs slow gait	Low ASM > Slow gait	5.02	0.004	0.056	0.053
Gait performance						
Gait cycle time	Robust vs slow gait	Slow gait > Robust	19.68	<0.001	<0.001	0.176
Gait cycle time	Robust vs low ASM	Low ASM > Robust	1.29	0.393	1.000	0.014
Gait cycle time	Low ASM vs slow gait	Slow gait > Low ASM	9.92	<0.001	0.006	0.097
Cadence	Robust vs slow gait	Robust > Slow gait	18.90	<0.001	0.002	0.170
Cadence	Robust vs low ASM	Robust > Low ASM	1.24	0.383	1.000	0.013
Cadence	Low ASM vs slow gait	Low ASM > Slow gait	9.52	0.001	0.008	0.094
Stance phase	Robust vs slow gait	Slow gait > Robust	3.72	0.113	0.506	0.039
Stance phase	Robust vs low ASM	Low ASM > Robust	0.06	0.766	1.000	0.001
Stance phase	Low ASM vs slow gait	Slow gait > Low ASM	2.48	0.101	0.506	0.026
Gait variability						

Outcome	Contrast	Direction	F	Permutation p	Holm p	ηp²
Gait cycle time CV	Robust vs slow gait	Slow gait > Robust	17.89	0.001	0.010	0.163
Gait cycle time CV	Robust vs low ASM	Low ASM > Robust	2.88	0.408	1.000	0.030
Gait cycle time CV	Low ASM vs slow gait	Slow gait > Low ASM	6.12	0.008	0.041	0.062
Stance phase CV	Robust vs slow gait	Slow gait > Robust	6.77	0.004	0.025	0.069
Stance phase CV	Robust vs low ASM	Low ASM > Robust	0.45	0.483	1.000	0.005
Stance phase CV	Low ASM vs slow gait	Slow gait > Low ASM	3.39	0.018	0.071	0.035
Swing phase CV	Robust vs slow gait	Slow gait > Robust	10.38	0.001	0.009	0.101
Swing phase CV	Robust vs low ASM	Low ASM > Robust	0.72	0.416	1.000	0.008
Swing phase CV	Low ASM vs slow gait	Slow gait > Low ASM	5.15	0.006	0.034	0.053

Exploratory localisation. Pairwise contrasts between phenotype groups from the Run A model on ranks (adjusted for stroke, sex, age, and height). Direction: the group with the higher covariate-adjusted mean rank of the outcome. p values by Freedman–Lane permutation (9999 permutations) restricted within matched sets, with a reduced model in which the two contrasted groups share one coefficient; Holm adjustment across all contrasts within a block (strength 21, gait performance 9, gait variability 9). F has 1 and 89 degrees of freedom for strength (n = 96) and 1 and 92 for the gait blocks (n = 99); ηp², partial eta-squared.

Table S15. Muscle quality: weaker-limb knee-extension strength per kilogram of appendicular skeletal muscle mass

(a) Cell medians by group and phenotype (N/kg)

Group	Phenotype	n	Median	IQR (Q1–Q3)
Stroke	Robust	7	9.68	8.45–11.45
Stroke	Slow gait	16	6.66	4.26–7.55
Stroke	Low ASM	7	10.84	8.40–13.25
Control	Robust	46	8.84	7.32–10.60
Control	Slow gait	6	5.78	4.80–8.65
Control	Low ASM	14	10.28	8.35–13.30

(b) Run A model on ranks: exposure terms and phenotype contrasts

Test	Direction	F (df1, df2)	Permutation p	Holm p	ηp²
Stroke	–	0.17 (1, 89)	0.615	–	0.002
Phenotype	–	11.78 (2, 89)	<0.001	–	0.209
Robust vs slow gait	Robust > Slow gait	14.72 (1, 89)	<0.001	0.001	0.142
Robust vs low ASM	Low ASM > Robust	1.63 (1, 89)	0.132	0.132	0.018
Low ASM vs slow gait	Low ASM > Slow gait	22.12 (1, 89)	<0.001	<0.001	0.199

Muscle quality = weaker-limb knee-extension strength (N) divided by appendicular skeletal muscle mass (kg). Run A model (stroke + phenotype + sex, age, height) on the ranked ratio; p values by Freedman–Lane permutation (9999 permutations) restricted within matched sets; Holm adjustment across the three contrasts; direction = the group with the higher adjusted mean rank. ASM (kg) was used as the denominator because segmental leg lean mass was not available for all participants; n = 96 (three unverifiable knee-extension values excluded).

Table S16. Software environment and data dictionary

(a) Software environment

Software	Version	Use
Python	3.12.3	Interpreter for the statistical analyses and for Figures 1–3
NumPy	2.4.4	Matrix computations for the multivariate tests; random numbers for the permutation tests
pandas	3.0.2	Data frames; reading the analysis data files
SciPy	1.15.3	Rank transformation; F-distribution p values; linear sum assignment for the matching
statsmodels	0.14.6	Holm adjustment; MANOVA cross-check; random-intercept models (Supplementary Table S10)
Matplotlib	3.10.9	Figures 1–3 (Figure 4: see note)
openpyxl	3.1.5	Reading Excel files: column headers checked for the muscle-quality denominator; gait-analysis exports read for Figure 2d
Pillow	12.2.0	Image handling in the figure scripts (Figure 2 illustration panels, image checks)
pydantic	2.13.4	Imported by the figure library used for the figures (figure-specification models)
Permutation test random seed	20260925 (master seed)	9,999 permutations per test, restricted to within matched sets (unrestricted in matched dataset A3, which has no matched-set identifier; Supplementary Table S9); per-test seed = first 8 hexadecimal digits of SHA-256 of "20260925|<test key>", read as an integer; NumPy default_rng (PCG64)

Python, NumPy, pandas, SciPy and statsmodels versions are those printed by the analysis scripts at run time; the other versions were read from the same environment, and the Matplotlib version is also recorded in the files of Figures 1–3. Figure 4 was drawn from the tabulated results in a separate Python 3.12.3 environment (Matplotlib 3.10.8, NumPy 2.4.2, pandas 3.0.3, Pillow 12.1.1, pydantic 2.13.4); no statistic was computed in that environment. The statistical analyses were run in Python only; no R, SPSS or Stata was used.

(b) Data dictionary

Variable	Definition	Unit / coding	Role in the analysis
Group	Stroke survivor or matched control	stroke / control	Exposure (stroke status, 2 levels) in all runs
Phenotype	Sarcopenia-related phenotype from ASM/Ht² and gait speed with the AWGS 2019 cut-offs: robust, ASM/Ht² at or above the sex-specific cut-off and gait speed at or above 1.0 m/s; slow gait with preserved muscle mass, ASM/Ht² at or above the cut-off and gait speed below 1.0 m/s; low ASM, ASM/Ht² below the cut-off regardless of gait speed	robust / slow gait with preserved muscle mass / low ASM	Exposure (3 levels) in Runs A, B and D and in the interaction run; removed in Run C; replaced by ASM/Ht² in Run E
Age	Age at assessment	years	Covariate in all runs; matching variable (±7-year caliper, Mahalanobis distance, signed age term)
Sex	Participant sex	M / F	Covariate in all runs; matched exactly; selects the sex-specific ASM/Ht² cut-off
Height	Body height	cm	Covariate in all runs; matching variable (Mahalanobis distance); converts ASM/Ht² to ASM (kg) for muscle quality
Weight	Body weight	kg	Matching variable (Mahalanobis distance); covariate in Run D

Variable	Definition	Unit / coding	Role in the analysis
ASM/Ht²	Appendicular skeletal muscle mass (multifrequency bioelectrical impedance analysis, InBody 370S) divided by squared height	kg/m²	Phenotype classification input (cut-offs below 7.0 kg/m² for men and below 5.7 kg/m² for women); continuous exposure in place of the phenotype in Run E
Gait speed	Mean gait speed across the valid gait cycles of the self-paced 6-min walk; mean of the two limbs	m/s	Phenotype classification input (cut-off 1.0 m/s); excluded from the gait performance block in the primary analysis; covariate in Run B; fourth outcome of the gait performance block in Run E
Hip flexion (iliopsoas)	Isometric maximal voluntary contraction (handheld dynamometer), maximum of three trials, tested seated with the hip and knee at 90°; weaker limb (smaller of the left and right values)	N	Outcome, strength block (all runs except Run B)
Hip extension (gluteus maximus)	Isometric maximal voluntary contraction (handheld dynamometer), maximum of three trials, tested prone; weaker limb (smaller of the left and right values)	N	Outcome, strength block (all runs except Run B)
Hip abduction (gluteus medius)	Isometric maximal voluntary contraction (handheld dynamometer), maximum of three trials, tested prone; weaker limb (smaller of the left and right values)	N	Outcome, strength block (all runs except Run B)
Knee flexion (hamstrings)	Isometric maximal voluntary contraction (handheld dynamometer), maximum of three trials, tested prone; weaker limb (smaller of the left and right values)	N	Outcome, strength block (all runs except Run B)
Knee extension (quadriceps)	Isometric maximal voluntary contraction (handheld dynamometer), maximum of three trials, tested seated with the hip and knee at 90°; weaker limb (smaller of the left and right values); three left-limb values in the stroke group could not be verified and are missing (n = 96)	N	Outcome, strength block (all runs except Run B; n = 96); omitted in the six-muscle sensitivity run (n = 99); numerator of muscle quality
Ankle plantarflexion	Isometric maximal voluntary contraction (handheld dynamometer), maximum of three trials, tested supine with the leg supported; weaker limb (smaller of the left and right values)	N	Outcome, strength block (all runs except Run B)
Ankle dorsiflexion	Isometric maximal voluntary contraction (handheld dynamometer), maximum of three trials, tested supine with the leg supported; weaker limb (smaller of the left and right values)	N	Outcome, strength block (all runs except Run B)
Gait cycle time	Mean duration of the gait cycle across the valid cycles of the self-paced 6-min walk (inertial sensors, Gait Analysis Toolkit v4.7.0); mean of the two limbs	s	Outcome, gait performance block (all runs)
Cadence	Steps per minute, mean across the valid cycles of the walk; mean of the two limbs	steps/min	Outcome, gait performance block (all runs)
Stance phase	Proportion of the gait cycle spent in stance, mean across the valid cycles of the walk; mean of the two limbs	%	Outcome, gait performance block (all runs)
CV of gait cycle time	Coefficient of variation of gait cycle time across the gait cycles of the walk (100 × SD / mean), for each limb; mean of the two limbs	%	Outcome, gait variability block (all runs)
CV of stance phase	Coefficient of variation of stance phase, for each limb; mean of the two limbs	%	Outcome, gait variability block (all runs)
CV of swing phase	Coefficient of variation of swing phase, for each limb; mean of the two limbs	%	Outcome, gait variability block (all runs)

Variable	Definition	Unit / coding	Role in the analysis
Muscle quality	Weaker-limb knee-extension strength (N) divided by appendicular skeletal muscle mass (kg), where ASM (kg) = $ASM/Ht^2 \times height^2$ (height in m)	N/kg	Outcome of the supplementary muscle-quality analysis (Run A model on ranks, n = 96; Supplementary Table S15)
Paretic side	Documented paretic side of stroke survivors (30 of 33)	L / R; NA if not documented and for controls	Defines the paretic-limb rule of the limb-rule analysis (Supplementary Table S13), under which controls contribute the mean of both limbs

The alternative limb rules in Supplementary Table S13 apply to the strength block: the stronger limb (larger of the left and right values) and the mean of both limbs, each with the seven muscle groups (n = 96), and the paretic limb for stroke survivors with a documented paretic side, with controls contributing the mean of both limbs, in 30 matched sets (n = 90) with six muscle groups (knee extension unavailable). Matched-set membership (one stroke survivor and two controls per set) is used to restrict the permutations and as the grouping factor of the random-intercept models (Supplementary Table S10), and is not listed. Body mass index, reported in Table 1, was calculated as weight divided by squared height (kg/m^2) and was used descriptively only.
